# Case Report: Lyme Borreliosis and Pregnancy - Our Experience

**DOI:** 10.3389/fmed.2022.816868

**Published:** 2022-03-28

**Authors:** Giusto Trevisan, Maurizio Ruscio, Nicola di Meo, Katiuscia Nan, Marina Cinco, Sara Trevisini, Patrizia Forgione, Serena Bonin

**Affiliations:** ^1^Department of Medical Sciences, University of Trieste, Trieste, Italy; ^2^Azienda Sanitaria Universitaria Integrata Giuliano Isontina, Trieste, Italy; ^3^Unità Operativa Semplice di Dermatologia, Centro Rif. Regionale Malattia di Hansen e Lyme, P.O. dei Pellegrini, ASL Napoli Centro, Naples, Italy

**Keywords:** pregnancy, Lyme borreliosis, antibiotic treatment, newborn, Borrelia, serology

## Abstract

Lyme Borreliosis (LB) is an infection transmitted by *Ixodes* sp. ticks. Its early manifestation includes erythema migrans rash. Since the discovery of LB in 1975, the question arose as to whether this infection could be vertically transmitted from mother to fetus during pregnancy, as transplacental transmission has already been known for other spirochetoses, such as syphilis, relapsing fever and leptospirosis. The first confirmed case with positive Lyme serology was described in 1985 in a 28-year- old mother who had acquired Lyme in the first trimester and then developed an erythema migrans rash. Subsequently, transmission of *Borrelia burgdorferi* sl. in humans from mother to fetus has been documented through identification of Borrelia spirochetes in fetal tissues/and or placenta by various methods including culture, PCR and indirect immunofluorescence. Adverse birth outcomes, which are limited in case of prompt LB treatment, included spontaneous miscarriage, preterm birth and hyperbilirubinemia, but also cardiac involvement and cutaneous angiomas have been documented although rarely. No significant associations were found between adverse outcomes at birth and the trimester of infection. Patients treated for gestational LB had a lower frequency of miscarriages and premature births, as also the frequency of congenital malformations was similar to that observed in the normal population. The recommended treatment for LB in pregnancy is Amoxicillin, 1 g 3 times a day for 14–21 days. In the present study, we report our case series, which includes 11 pregnant women, 6 of which developed erythema migrans during pregnancy (between week 8 and 34), 3 had myoarticular or neurological symptoms and 2 had positive serology, but did not develop any clinical symptoms. Our data stress on the importance of early antibiotic treatment also in seropositive gestating women without symptoms in order to avoid any possible complication to fetus and newborns.

## Introduction

A wide range of infectious diseases can occur in pregnancy. Their acquisition, clinical presentation and course during gestation can be altered due to impaired maternal immunity. Not all, but some infectious diseases, of viral, fungal, bacterial origins, including Lyme disease and protozoal borreliosis, can lead to serious consequences to mother and/or fetus (1). Spirochaetes, both those transmitted by ticks and by other routes, usually have vertical transmission in pregnancy and cause fetal damage. Notably, for some Borreliae of the relapsing fever group (RF) transmitted by the soft ticks *Ornithodoros* sp., a vertical transmission is reported (2, 3). RF Borreliae are known to cause abortion in animals, in particular in cattle (*Borrelia coriaceae* is the agent of epizootic bovine abortion in the USA) and in horses (equine abortion) as documented in the USA (4) and in Iran and Israel (5).

Lyme borreliosis (LB) is an infection transmitted by *Ixodes* sp ticks, where vertical transmission of Borreliae seems to be negligible (6). The early manifestation of LB in humans is erythema migrans. Since its discovery in 1975, the question arose as to whether this infection could be transmitted to the fetus during pregnancy (7). Transplacental transmission in humans was indeed reported for other spirochaetosis, such as syphilis, RF and leptospirosis (8, 9) (Table 1).

**Table 1:** Vertical transmission of borreliae.

**Borrelia group**	**In ticks**	**In animals**	**In humans**
Lyme group	Rare*	Yes	Yes
Echidna-reptile group	Unknown	Unknown	Unknown**
Relapsing fever group	Yes	Yes	Yes

*The vertical transmission of Borrelia burgdorferi s.l. in Ixodes sp. ticks does not normally occur, however rare cases of vertical transmission have been documented. **There is no evidence of infection in humans.

Vertical transmission of LB was suspected in 1983 in a woman with febrile illness during pregnancy, but serological analyses for Lyme or syphilis were not carried out (10).

The first confirmed case of LB in a pregnant woman was described in 1985 in a 28-year- old mother who had acquired LB with erythema migrans in the first trimester and delivered at 35 weeks. The mother developed symptoms consistent with LB post-delivery as documented by positive immunofluorescence assay for LB. The child died of congenital heart disease and the autopsy revealed spirochetes infiltrating the spleen, kidneys, and bone marrow, but not cardiac tissue (11).

Lyme Group Borreliae can be transmitted vertically in specific animals which act as reservoirs and in humans (Table 1). Therefore, *Borrelia burgdorferi* sensu lato might be naturally maintained in an enzootic cycle by transplacental transmission (12).

In 1986 MacDonald described fetal LB, with cardiac involvement and fetal death (13). The different signs and symptoms in gestational Lyme borreliosis mirror the difference found in prenatal syphilis. The transplacental transmission of the spirochaete from mother to fetus is indeed possible. Clinical studies, including autopsy data have associated gestational Lyme borreliosis with different medical concerns including fetal death, hydrocephalus, cardiovascular abnormalities, neonatal respiratory distress, hyperbilirubinemia, intrauterine growth retardation, cortical blindness, sudden infant death syndrome and maternal toxemia in pregnancy (13).

The abovementioned birth outcomes would support a vertical transmission of *B*. *burgdorferi*, with possible negative consequences for the fetus (14, 15).

Overall, it seems that adverse birth outcomes in gestational LB are more likely if LB is not treated.

The initiation of antibiotic treatment in some spirochaetal infections can cause Jarisch-Herxeimer Reaction (JHR), which is a transient clinical phenomenon manifesting within 24 h of antibiotic therapy with fever, chills, rigors, nausea and vomiting, headache, tachycardia, hypotension, hyperventilation, flushing, myalgia, and exacerbation of skin lesions (16). JHR can also occur in pregnancy, however in LB it is not frequent (17) and less severe than observed for other spirochaetosis (18).

In the present study we describe our experience regarding LB in pregnancy.

## Clinical Cases

**Figure 1:**
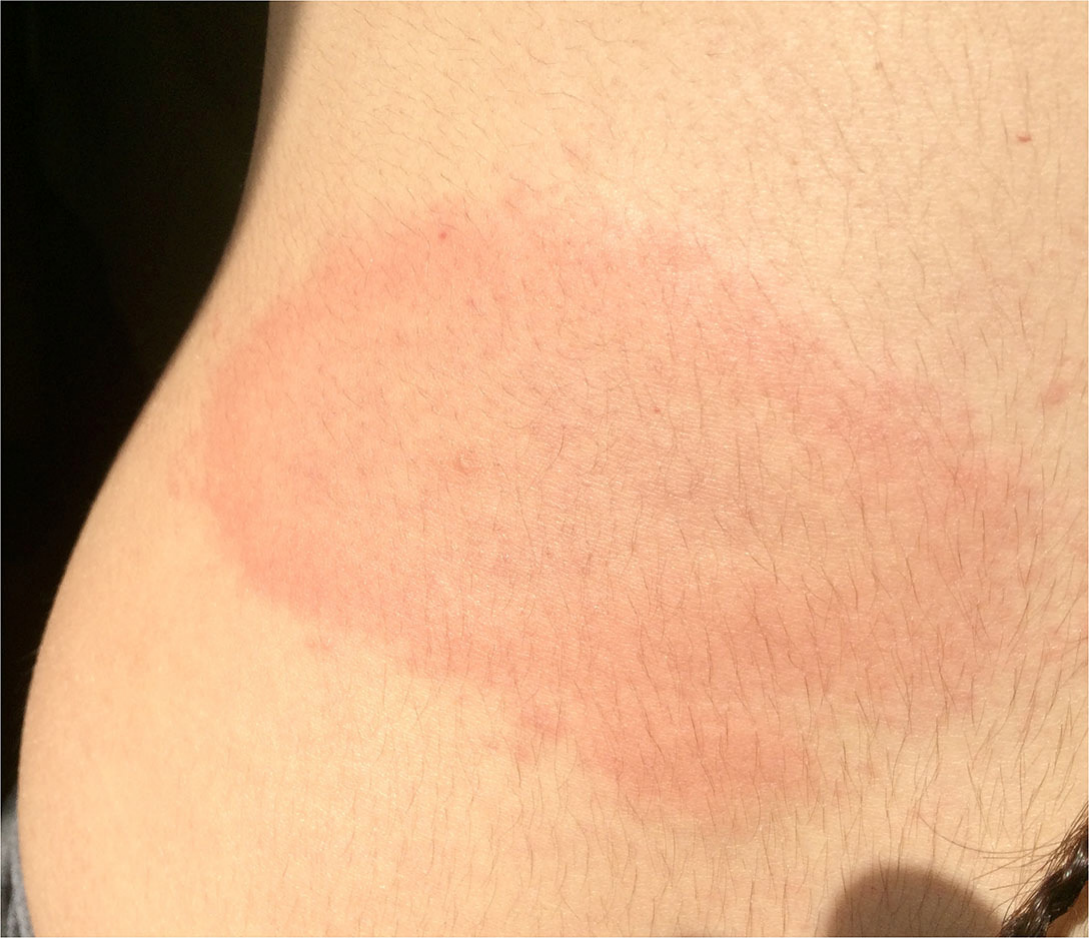
Eythema migrans of the back developed in the first trimester of pregnancy (Case 7).

From 2008 to 2020, 11 pregnant women with LB were followed at Trieste University Hospital. Mean age of mothers at time of delivery was 30.5 years (range 21–40). The clinical data are summarized in Table 2. In short, 5 women were diagnosed and treated for LB in the first trimester, 3 in the second trimester and the last 3 women in the third trimester of pregnancy. Six patients developed erythema migrans during pregnancy (Figure 1), three had migratory myoarticular involvement and one of them also presented neurological manifestations. In two women serology tests showed high levels of IgG antibodies for LB despite patients not presenting any clinical symptom. Serological test was negative only in a 26-year-old patient with erythema migrans.

**Table 2:** Cases observed from 2008 to 2020.

**Case**	**Age**	**Tick bite**	**Week of pregnancy**	**Clinical manifestations**	**Blood borrelia tests**	**Treatment**	**JHR**	**Newborn at birth**	**After 1 year**
1	40	Yes	15 2 Trim	Erythema migrans thigh Urticaria	IFA IgM = 1:64 IgG = 1:512	Amoxicillin 1 gr 3x/day for 14 days	No	Healthy	Healthy Growth and weight according to age
2	21	Yes	8 1 Trim	Migratory arthralgias Neurological disorders	ELISA IgG neg IgM pos WB IgG-IgM Osp C + Blood PCR+	Amoxicillin 1 gr 3x/day for 14 days After 6 months Neurological disorders Ceftriaxone 2 gr iv/die x 21 days 1 year after Healthy and Abs neg	No	Healthy	Healthy Growth and weight according to age
3	31	Unknown	34 3 Trim	Erythema migrans	ELISA IgG neg IgM pos WB IgG-IgM Osp C +	Amoxicillin 1 gr 3x/day for 14 days	No	Healthy	Healthy Growth and weight according to age
4	30	Yes	24 2 Trim	Erythema migrans back	ELISA IgG + IgM +	Amoxicillin 1 gr 3x/day for 14 days	No	Healthy	Healthy Growth and weight according to age
5	37	Unknown	16 2 Trim	Migratory arthralgias	ELISA IgG- IgM+ Western Blot IgG- IgM OspC+	Amoxicillin 1 gr 3x/day for 14 days	No	Healthy	Healthy Growth and weight according to age
6	32	Yes	28 3 Trim	None	ELISA IgG+ IgM+ Western Blot IgG+ IgM OspC+	Amoxicillin 1 gr 3x/day for 14 days	No	Healthy	Healthy Growth and weight according to age
7	30	Yes	10 1 Trim	Erythema migrans	ELISA IgG- IgM+ Western Blot IgG- IgM OspC+	Amoxicillin 1 gr 3x/day for 14 days	No	Urgent cesarean birth at month 7 of pregnancy, healthy, ECG normal	Healthy Growth and weight according to age
8	31	Yes	9 1 Trim	Erythema migrans	ELISA IgG- IgM+ Western Blot IgG- IgM OspC+	Amoxicillin 1 gr 3x/day for 14 days	No	Healthy	Healthy Growth and weight according to age
9	27	Unknown	7 1 Trim	Left Knee Arthritis, Headache. Low-grade Fever	CLIA IgG>240 IgM+ Western Blot IgGVlsE+ IgM OspC+	Amoxicillin 1 gr 3x/day for 14 days	No	Angiomatous patches	Healthy Growth and weight according to age Angiomatous Patches *
10	26	Yes	30 3 Trim	Erythema migrans leg		Amoxicillin 1 gr 3x/day for 14 days	No	Healthy	Healthy Growth and weight according to age
11	30	Yes	11 1 Trim	None	CLIA IgG+ IgM+ Seroconversion	Amoxicillin 1 gr 3x/day for 14 days	No	Healthy	Healthy Growth and weight according to age

*Angiomatous patches resolved within 2 years as in the 2 years examination they were disappeared.

**Figure 2:**
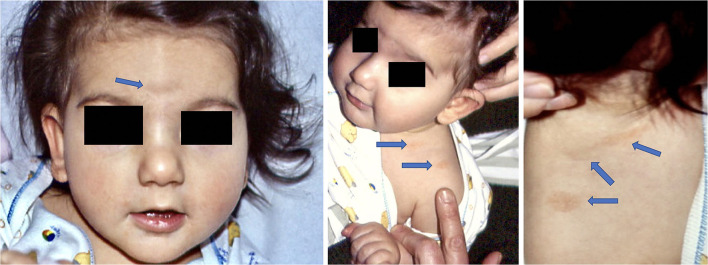
Child born with angiomatous patches at 7 months (Case 9). At 1 year examination angiomatous lesions were still visible, but not at 2 years follow-up.

All women were immediately treated at the time of diagnosis with Amoxicillin 1g 3x/day for 14 days. In one case (case 7) the baby was born prematurely at 7 months by an emergency cesarean section, and in one case the newborn (case 9) presented angiomatoid patches (Figure 2), in the center of its forehead, on its left shoulder and on its back, which regressed spontaneously 18 months later. The other 8 babies were born at full term and healthy. At follow-up, after 1 year, most mothers and all babies were healthy and the weight and length of the premature baby were also appropriate for its age.

At the time of diagnosis, the 21-year-old woman (case 2) with articular and neurological involvement had positive IgM antibodies against *Borrelia burgorferi* both in blood and CSF and positive PCR results for Borrelia in DNA isolated from her blood. The patient improved partially after treatment with Amoxicillin during pregnancy. Two years later, because of persistent symptoms, she was treated with Ceftriaxone 2 gr iv/day for 21 days. After one additional round of treatment 6 months later she was clinically healed and her serology was negative for Borrelia.

## Discussion

Pregnant women are considered a vulnerable group due to a weakened immune system. Therefore, they are more susceptible to infection with an increased risk for severe illness. Furthermore, infections in pregnant women have the added gravity of potential infection in the developing fetus which may have important consequences including death *in-utero* or lifelong debilitation. Taking this evidence into consideration, women are at a higher risk of developing LB and coinfections during pregnancy (19). Complications of maternal Lyme borreliosis in pregnancy outcomes have been already reported (20), including stillbirth and possible congenital malformations (21). The vertical transmission of Lyme group Borreilae has been supported by the detection of spirochaetemia (11, 22) or erythema migrans rash (23) in newborns. Furthermore, also Borrelia specific antibodies have been detected in the cerebrospinal fluid of an infant with documented neurologic dysfunction (24). Therefore, gestating women with LB can transmit the disease to fetus with possible adverse outcomes, especially if antibiotic therapy is not carried out in women. Nevertheless, it is not yet well established if there is a greater risk for the fetus in pregnant women in endemic areas for LB. Some authors reported a higher incidence of abortions in the Italian region Friuli-Venezia Giulia, which is endemic for LB (25). In the LB endemic area of Westchester, New York, US, a higher incidence of cardiac abnormalities in newborn in comparison to the population of non-endemic areas was recorded (26). However, Strobino and co-workers did not observe any significant difference in fetal deaths, pre-term delivery, congenital anomalies and adverse outcomes in endemic vs. non-endemic population for LB (21).

Tick bite or suspected LB should be managed similarly in pregnant and non-pregnant adults, including antibiotic therapy for prophylaxis and treatment (27). Nonetheless, greater attention in pregnancy should be given to the possible symptoms of infection, which should be accompanied by a prompt investigation for Lyme Borrelia (7) and other infections transmitted by Ixodes ticks. Although in our case series we did not detect any other co-infecting bacteria or viruses, there are indeed scientific records reporting on Rickettsia (28), Babesia (29, 30) and Tick-Borne Encephalitis (TBE) virus (31, 32) in pregnant women. Co-infecting agents can be harmful *per se* to gestating women and fetuses. Among them, Babesia can be vertically transmitted (29, 33) and was diagnosed after antibiotic treatment for LB in a pregnant woman because of an acute hemolysis event, which resolved after proper antibiotic treatment without any consequence for mother and newborn (30). Transplacental transmission in mammals and humans has also been documented for *Anaplasma phagocytophilum* (34). Data are quite different for the TBE virus transmitted by Ixodes ticks and affecting the central nervous system. There are very few reports of TBE in pregnancy and clinical as well as virological data suggest that fetal TBE infection did not occur, despite severe manifestations in pregnant women (31, 32).

The rate of infected ticks varies in endemic areas from 10 to 18% with an average incidence of Borrelia in *Ixodes scapularis* in the US of around 11.2%; in *Ixodes ricinus* in Latvia of around 18%, in Germany of 12.8% and in Sweden of 10–15% (35–37). The incidence of LB or seroconversion after tick-bite in Northern European countries was 5% (78/1,546), of which 33 (2%) developed LB, while 45 subjects (3%) seroconverted only (38). Therefore, for Borrelia infected ticks the risk of infection is ~7–10 times higher, justifying preventive antibiotic treatment with Amoxicillin after a bite of a Borrelia positive tick, especially in pregnancy.

In addition to the Lyme group Borreliae, Ixodes ticks can also transmit another type of Borrelia, named *Borrelia miyamotoi*, which is a member of the relapsing fever (RF) group. For RF Borreliae the transmission in pregnancy as well as fetal damage and abortion in humans and animals are well known (39, 40). Infections by RF Borreliae in pregnant hosts can be also complicated by Jarisch-Herxheimer reaction, which has also been reported for other spirochaetes and may impair fetus life. However, JHR has a minor impact in LB infections (41).

In our case series, 8 patients treated with Amoxicillin did not have any complications neither for the mothers nor the newborns. We acknowledge however that our sample size is limited to conclusively ascertain the impact of treatment on the prevention of adverse fetal outcome, in larger studies comorbidity and maternal age should also be considered as possible factors impacting both on the course of LB and outcome. In our series patients did not report any comorbidity, despite a mild atopy in patient 1. Nevertheless, it is possible that coexistent comorbid conditions in pregnant women could alter the course of LB even if treated, as shown in immunocompromised patients (42). Nevertheless, at present there are no reports describing the course of LB and outcome in pregnant women with comorbidities. Although in our case series two mothers were over 35 years their clinical course and outcome were similar to those reported for younger women. However, we cannot exclude that advanced maternal age could influence the course of LB in pregnancy, because of the observed comorbidities as well as complications during pregnancy, delivery and outcome in women aged over 35 (43). In our series, an emergency cesarean section was performed and the child was born with erythematous angiomatous patches which disappeared spontaneously within 24 months, in line with the resolution of this manifestation. We acknowledge however that we cannot prove that those patches were caused by LB in the mother during pregnancy, although other authors reported angiomas in children born from mothers with LB (26, 44). In none of our cases JHR was observed after the initiation of treatment with Amoxicillin. A prompt antibiotic therapy with Amoxicillin during pregnancy prevented in most of our patients and newborns any type of complication in agreement with Waddel and co-authors who reported significantly fewer adverse birth outcomes in women treated for gestational LB (45). However, Maraspin et al. had similar results in 7 pregnant women treated with ceftriaxone for 14 days (46), recommending in addition to the above mentioned treatment in pregnant women with LB intravenous antibiotic therapy with penicillin, not only for early disseminated disease, but also for solitary erythema migrans (47).

In our case series no associations have been found between the trimester of pregnancy and LB manifestations, as already reported (45). Nevertheless, arthropod-borne bacterial diseases are difficult diagnoses in pregnancy, because they can mimic many other pathologic conditions and common pregnancy-specific conditions, such as typical and atypical preeclampsia, or symptoms of pregnancy itself (48). In addition, as shown in our case series, some women can have positive serology for LB, without evident clinical symptoms. This poses the question if in endemic areas for Lyme Borreliosis, serological tests for Borrelia should be included routinely for pregnant women to prevent possible adverse effect, as suggested for syphilis (49). Although, a positive serology does not prove an active LB without clinical symptoms, we should acknowledge that even in those cases a possible transmission to the fetus should be avoided with an antibiotic prophylaxis. Currently there is paucity of data surrounding tick-borne infections in pregnancy and long-term outcomes for mother and infant for conditions like LB and co-infections. At present there are no established international surveillance systems to identify and gain understanding of these infections in pregnancy (50) and international guidelines to manage gestational LB in endemic area are not available.

Taken that there is not increased risk to pregnant women who develop LB if they receive appropriate antimicrobial therapy (51) and that in treated gestating women the frequency of premature births and congenital malformations is similar to that observed in the general population (52), an antibiotic prophylaxis in seropositive gestating women without symptoms could be stressed or at least considered in endemic areas for LB. In pregnant women in endemic areas for Lyme Borreliosis, also testing antibodies against Borrelia should be routinely recommended (53), in case of positivity the same analysis could also be suggested on umbilical cord blood at delivery. Pregnant women with LB should be treated and clinically followed, with particular attention for cutaneous, myoarticular, neurological, ocular and cardiac manifestations, which could require a fetal ECO-cardiography follow-up. Newborns should be examined for possible clinical manifestations, as babies born from mothers with gestational LB have been documented in some cases to be small for dates, or presenting several manifestations including pyloric stenosis, cutaneous annular erythemato-papular eruption, cutaneous angiomas, neurological disorders, muscle hypotonia, hypospadias and skeletal abnormality (26, 44).

## Data Availability Statement

The original contributions presented in the study are included in the article, further inquiries can be directed to the corresponding author.

## Ethics Statement

Ethical review and approval was not required for this study in accordance with the local legislation and institutional requirements. Written informed consent to participate in this study was provided by the participants or their legal guardian/next of kin. Written informed consent was obtained from the individual(s), and minor(s)' legal guardian/next of kin, for the publication of any potentially identifiable images or data included in this article.

## Author Contributions

GT and SB: conceptualization and writing-original draft preparation. GT, MR, NM, KN, ST, and SB: methodology. GT, MR, NM, KN, and ST: data collection. GT, MR, NM, KN, ST, and PF: data analysis. ST, NM, KN, and PF: writing-review and editing. GT: supervision. All authors have read and agreed to the published version of the manuscript.

## Funding

The APC to publish this article was funded by the Associazione Lyme Italia e Coinfezioni.

## Conflict of Interest

The authors declare that the research was conducted in the absence of any commercial or financial relationships that could be construed as a potential conflict of interest.

## Publisher's Note

All claims expressed in this article are solely those of the authors and do not necessarily represent those of their affiliated organizations, or those of the publisher, the editors and the reviewers. Any product that may be evaluated in this article, or claim that may be made by its manufacturer, is not guaranteed or endorsed by the publisher.
